# Relationship between Circadian Phase Delay without Morning Light and Phase Advance by Bright Light Exposure the Following Morning

**DOI:** 10.3390/clockssleep5040041

**Published:** 2023-10-23

**Authors:** Michihiro Ohashi, Taisuke Eto, Toaki Takasu, Yuki Motomura, Shigekazu Higuchi

**Affiliations:** 1 Graduate School of Integrated Frontier Sciences, Kyushu University, Fukuoka 815-8540, Japan; ohashi.michihiro.572@s.kyushu-u.ac.jp (M.O.);; 2 Research Fellow of the Japan Society for the Promotion of Science, Fukuoka 815-8540, Japan; 3 Department of Human Life Design and Science, Faculty of Design, Kyushu University, Fukuoka 815-8540, Japan; 4 Department of Sleep-Wake Disorders, National Institute of Mental Health, National Center of Neurology and Psychiatry, Tokyo 187-8551, Japan

**Keywords:** circadian phase, photoentrainment, light, dim light melatonin onset, sleep

## Abstract

Humans have a circadian rhythm for which the period varies among individuals. In the present study, we investigated the amount of natural phase delay of circadian rhythms after spending a day under dim light (Day 1 to Day 2) and the amount of phase advance due to light exposure (8000 lx, 4100 K) the following morning (Day 2 to Day 3). The relationships of the phase shifts with the circadian phase, chronotype and sleep habits were also investigated. Dim light melatonin onset (DLMO) was investigated as a circadian phase marker on each day. In the 27 individuals used for the analysis, DLMO was delayed significantly (−0.24 ± 0.33 h, *p* < 0.01) from Day 1 to Day 2 and DLMO was advanced significantly (0.18 ± 0.36 h, *p* < 0.05) from Day 2 to Day 3. There was a significant correlation between phase shifts, with subjects who had a greater phase delay in the dim environment having a greater phase advance by light exposure (r = −0.43, *p* < 0.05). However, no significant correlations with circadian phase, chronotype or sleep habits were found. These phase shifts may reflect the stability of the phase, but do not account for an individual’s chronotype-related indicators.

## 1. Introduction

Mammals, including humans, have an internal clock (circadian rhythm) with an approximately 24 h period, and the principal circadian clock of the brain is located in the suprachiasmatic nucleus (SCN) of the hypothalamus [1]. This clock oscillates autonomously in the absence of time cues from external stimuli due to a negative feedback loop via transcription and translation of a group of genes called clock genes [2]. These circadian rhythms are synchronized to environmental light-dark cycles. Light is the strongest entrainment cue of circadian rhythm [3], and light information from photoreceptor and ganglion cells in the retina is transmitted via the retinohypothalamic tract (RHT) [4], which controls the phase of the circadian rhythm by conveying information about the light and darkness of the external world to the center of the clock.

In a constant dim light environment without a light-dark cycle, the phase of the circadian rhythm gradually deviates from the initial phase [5]. The amount of phase drift under dim light exceeds a maximum of 1 h per day in a constant routine protocol [6,7]. Although this is significantly correlated with the individual’s circadian period, the inter-individual variation in the amount of deviation has been reported to be greater than that in the circadian period, and it is 2.6 times the variation in the difference between the circadian period and 24 h [7]. Because the circadian period is thought to be slightly longer than 24 h, the circadian phase is easily delayed [8,9,10]. Therefore, in order to entrain to the light-dark cycle of the external world, the phase of the circadian rhythm must be entrained to 24 h by the phase advance effect of light. However, the amount of phase advance, i.e., difference between the circadian period and 24 h, required for entrainment varies among individuals [11]. The phase response of circadian rhythms to light depends on light intensity [12], duration [13], spectral characteristics [14,15], and timing of light exposure. The phase response curve (PRC) is defined as a curve with the circadian phase of the stimulus exposure on the horizontal axis and the stimulus-induced phase shift on the vertical axis. Many previous studies on light-induced PRCs in humans showed that subjective morning light advances the phase of circadian rhythms and subjective night light delays the phase of circadian rhythms [16,17,18,19]. Even though previous studies showed that there are individual differences in the non-visual effects of light [20,21,22,23,24], there have been few studies in which the individual differences in the effect of phase advance due to morning light were examined. As noted above, morning light exposure advances (resets) the circadian phase, which tends to be delayed under dim light, so the individual differences in phase advance by morning light exposure and phase drift under dim light may be related to each other.

Some previous studies have shown associations between the circadian period and chronotype or phase angles (phase relationship between sleep timing and circadian timing). Those studies showed that individuals with longer circadian periods are often later chronotypes or have smaller phase angles (later circadian phase relative to sleep timing) [6,7,8,9,25,26]. If natural phase delay (phase delay under dim light excluding the effects of phase shift by light) reflects on the circadian period, individuals with a greater natural phase delay may have entrainment of the circadian phase at a later time and they may have a nocturnal chronotype, later sleep habits or smaller phase angles. On the other hand, mathematical models have shown that if the amount of phase advance due to light in the morning is great, the circadian phase will entrain at an earlier time [27]. In other words, individuals with a greater amount of phase advance due to morning light may be more likely to have a phase advance and be a morning person.

In the present study, we recruited 28 subjects and investigated the individual differences in the amount of natural phase delay of circadian rhythms after spending a day under dim light (from Day 1 to Day 2) and in the amount of phase advance due to bright light exposure for 1 h the following morning (from Day 2 to Day 3). The main objective of this study was to examine the relationship between the phase shift by morning light exposure and that under dim light and to test the hypothesis that individuals with greater phase delay under dim light would have greater phase advance due to morning light. As a secondary objective, we also examined the relationships of these phase shifts with chronotype-related indicators (circadian phase, chronotype, sleep habits, and phase angles) to test whether these phase shifts explain features by chronotypes. In addition, to evaluate the effect of circadian phase with light exposure, we also examined the relationship between the circadian time at which light exposure was initiated and the amount of phase shift due to morning light.

## 2. Results

### 2.1. Amounts of DLMO Shifts

**Figure 1:**
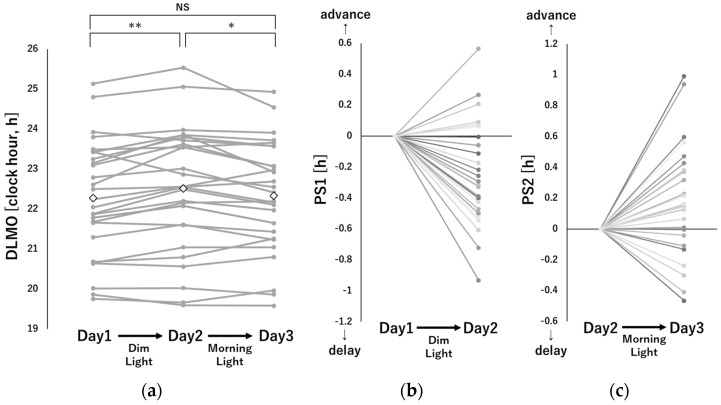
Individual data of absolute DLMO for (**a**) Day 1 to Day 2 (PS1, (**b**)) and Day 2 to Day 3 (PS2, (**c**)). Black-framed squares indicate DLMO averages for each day (NS: not significant, *: *p* < 0.05, **: *p* < 0.01).

Figure 1 shows the dim light melatonin onset (DLMO) shift for each subject and Figure S1 shows individual melatonin concentration profiles used in the analysis. DLMO showed a significant delay from Day 1 to Day 2 (Phase shift 1; PS1) under dim light (−0.24 ± 0.33 h, *p* < 0.01), while DLMO was significantly advanced from Day 2 to Day 3 (Phase shift 2; PS2) by the bright morning light exposure (0.18 ± 0.36 h, *p* < 0.05). There was no significant difference between the DLMO on Day 1 and that on Day 3 (−0.05 ± 0.37 h, *p* = 0.46). No significant sex differences were identified in Day1 DLMO (22:13 ± 1:19 vs. 22:19 ± 1:35, *p* = 0.86), PS1 (−0.25 ± 0.29 vs. −0.23 ± 0.37 h, *p* = 0.90) and PS2 (0.09 ± 0.29 vs. 0.26 ± 0.39 h, *p* = 0.20) (male vs. female, respectively).

### 2.2. Natural DLMO Delay (PS1) and DLMO Advance by Morning Light (PS2)

**Figure 2:**
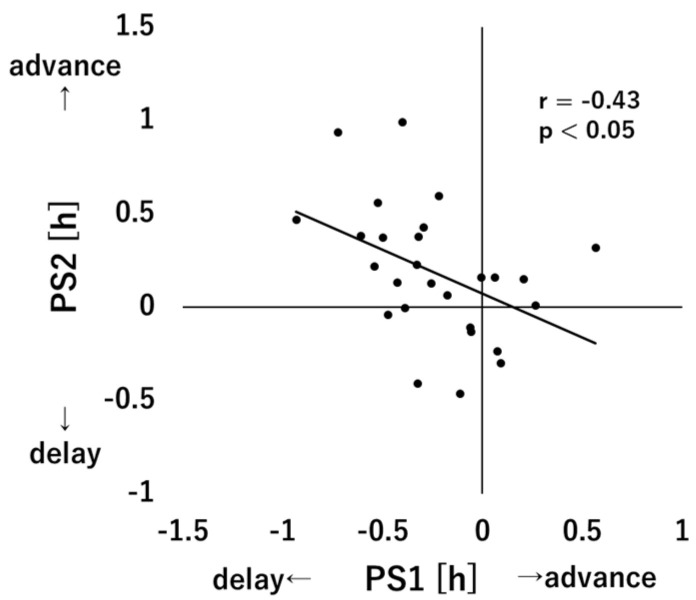
Correlation between PS1 and PS2. The points indicate individual data and the black line indicates the regression line.

Figure 2 shows the relationship between PS1 and PS2. A significant correlation was found between PS1 and PS2 (r = −0.43, *p* < 0.05), with subjects who showed greater delay in PS1 showing greater advance in PS2.

### 2.3. DLMO Shifts and Chronotype-Related Indicators

Table 1 shows the means and standard deviations of chronotype-related indicators. The relationships of the chronotype-related indicators with the DLMO shift from Day 1 to Day 2 (Phase Shift 1; PS1) and that from Day 2 to Day 3 (Phase Shift 1; PS2) are also shown. None of the correlation coefficients (r-values) of the indicators were significant after FDR correction. Sleep onset time and wake time during the sleep reporting period were 1:30 ± 1:17 and 8:31 ± 1:10, respectively. These showed a high consistency with sleep onset time (sleep onset weekday: r = 0.74, sleep onset free day: r = 0.81) and wake time (wake time weekday: r = 0.76, wake time free day: r = 0.83) in MCTQ coefficients. No significant correlations with PS1 (sleep onset time: r = −0.16, wake time: r = 0.19) or PS2 (sleep onset time: r = 0.32, wake time: r = 0.28) were identified.

### 2.4. Circadian Time of Light Exposure and DLMO Advance by Morning Light (PS2)

**Figure 3:**
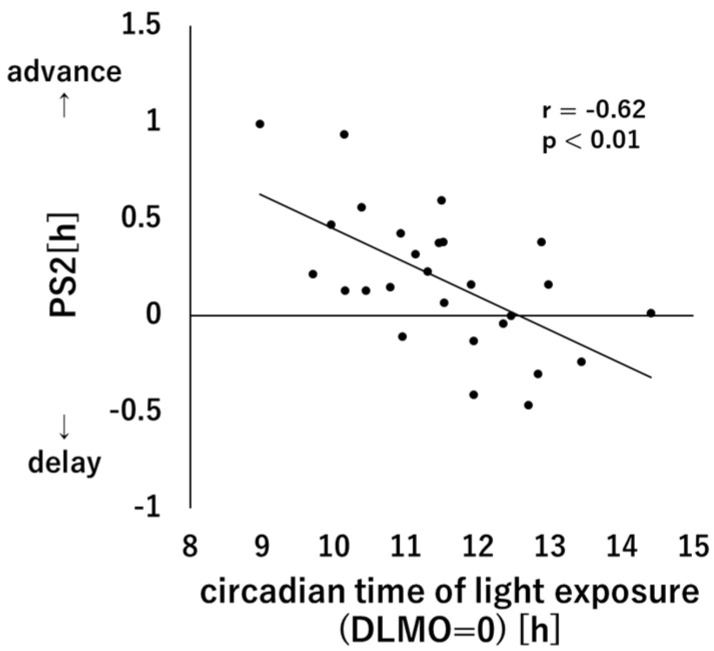
Correlation between circadian time of light exposure (start time) and PS2. The points indicate individual data and the black line indicates the regression line.

Figure 3 shows the relationship between circadian time of light exposure (start time) and PS2. A significant correlation was found between circadian time of light exposure and PS2 (r = −0.62, *p* < 0.01), with subjects who showed early circadian time of light exposure showing greater advance in PS2. Partial correlation analysis of PS1 and PS2 with control for circadian time of light exposure showed that the partial correlation coefficients between PS1 and PS2 were not significant (r = −0.18, *p* = 0.37).

## 3. Discussion

A significant natural phase delay under dim light was found in this study. This finding is consistent with results of previous studies showing a significant phase delay in dim environments [5,6,7,10] and it may reflect circadian periods that are longer than 24 h at the group scale [8,9,10]. However, there were also large individual differences in the amount of this phase delay. We expected that these individual differences would be associated with chronotype-related indicators, but no such correlations were found. If a significant correlation had been found, as has been observed in previous studies about the circadian period [6,7,8,9,25,26], it would indicate that the present natural phase delay may reflect the circadian period and define the chronotype of individuals, but this was not possible in the present study. One possible reason for this is that this natural phase delay may include other factors such as scalloping effects [28]. Various human circadian periods including 24.18 ± 0.13 h [10], 24.22 ± 0.22 h [8] and 24.22 ± 0.24 h [9] have been reported. The amount of natural phase delay under dim light identified in this study (−0.24 ± 0.33 h) is similar to the differences between the endogenous period and 24 h found in previous studies. However, the range and SD of the results were larger than those of the circadian period found in previous studies [8,9,10]. The amount of its deviation is similar to the variation in the amount of daily drift in a constant routine protocol [6,7]. Wright et al. [7] reported that the amount of phase drift per day during a constant routine protocol in a dim environment was significantly correlated with individual differences in the circadian period as measured by a subsequent forced desynchronization protocol, but the coefficient of determination was about only 41%. At least several days of experimentation would be needed to reduce the error. Another possible reason for the inconsistent results regarding the association with the circadian period is differences in the characteristics of the populations used as subjects. Even if the natural phase delay reflected the circadian period in this study, it is possible that a significant correlation was difficult to be obtained because chronotype-related indicators are restricted by social factors [29] and future studies with subjects other than university students may be necessary. In addition, although the subjects in this study had a variety of sleep habits, there was no extreme morningness or eveningness according to the MEQ. The lack of extreme chronotypes may be one of the reasons why significant correlations could not be confirmed.

A significant phase advance was also observed in the phase shift caused by morning light. It was expected that, as with the amount of natural phase delay, there would be associations with individual chronotype-related indicators, but no such correlation was found. A previous study showed that patients with delayed sleep-wake phase disorder (DSWPD) had a greater amount of phase delay due to night light exposure than did healthy controls [30]. This is reasonable and is consistent with the results of mathematical models showing that the greater the light-induced phase delay is, the later is the timing of photoentrainment of the circadian rhythm, due to the shape characteristics of the PRC and differences in photosensitivity [27]. This idea applies to morning light as well, and the intensity of the phase advance due to morning light may be related to the chronotype and a great phase advance effect due to morning light may make individuals more likely to become morning persons. However, in the present study there was no such correlation and no results supporting this idea were obtained.

Phase advance due to morning light was significantly correlated with natural phase delay, and the greater the natural phase delay was, the greater was the light-induced phase advance. As a result, there was no difference between DLMO on Day 1 and that on Day 3 in this study. Although the phase delay and phase advance cancelled each other on average, the relationship was also maintained within individuals. The fact that neither individual differences in natural phase delay nor individual differences in morning light-induced phase advance correlated with chronotype-related indicators may be due to a negative correlation between the two-phase shift quantities, and even if morning light exposure was associated with morning type and natural phase delay was associated with night type, the association between phase shift and chronotype may have been cancelled out.

Humanity was born in an era without artificial lighting. In such an environment, the circadian phase may have been balanced by natural phase delay and phase advance by sunlight. However, with the spread of artificial lighting, we are now influenced by artificial night light. As a result, the balance may have been disturbed and we may have become more nocturnal [31,32,33]. In that case, it would be even more important to strengthen the circadian phase advance effect and to balance against the influence of night light [34,35,36]. Moreover, the sensitivity to light at night may be a major factor in the nocturnal shift of our circadian phase. Photosensitivity and nocturnalization of circadian rhythms have been examined in terms of melatonin suppression as well as phase delay due to light at night [37,38]. Therefore, nighttime light sensitivity and its relationship to chronotype need to be investigated in future studies.

In this study, we tried to set the timing of light exposure at the same circadian time as much as possible, but there was in fact a gap of about ± 1.3 h (SD). Although we thought that such a small gap would not make much difference according to a past PRC study using 1-h light exposure [19], the actual results showed that the individual differences in phase advance due to morning light were significantly correlated with the circadian time of light exposure and that the phase advance was greater for those who started light exposure earlier in the circadian time as shown in Figure 3. The past PRC study by St. Hilaire et al. [19] had a small number of subjects and the data to verify individual differences were not available. Moreover, the reproducibility of the shape of the curve itself may be poor due to the large inter-individual variability in the same circadian timing. The results of the present study suggest that even 1 h of light exposure may show a clear peak of amplitude in the phase advance zone of the PRC. This negative correlation was also seen in a previous study in which melatonin administration was combined with light exposure [34] and is consistent with the results of a previous study on PRCs with 6.7 h of light exposure [16]. In a study using light at night by Watson et al. [30], the time of light exposure varied among individuals, and the individual differences were therefore controlled by using the relative phase shift computed by dividing an individual’s observed phase shift by the predicted phase shift according to past studies. However, in the present study, since a linear relationship was found between the two variables, partial correlation analysis was performed with control of the effect of individual differences in circadian time of light exposure. As a result, the relationship between phase advance and natural phase delay disappeared. Therefore, the timing of light exposure may mediate the relationship between these two variables, and further examination is needed to clarify these relationships.

This study has some limitations. First, the amount of natural phase delay in the dim light and the amount of phase advance due to light in the morning were each measured only once. Although the circadian period is highly reproducible within individuals [39], the possibility that the amount of natural phase delay in the dim environment does not strongly reflect that the circadian period itself was mentioned above. Additional studies with measurement of natural phase delays on consecutive days or paced over a period of time should be considered to confirm the intra-individual reproducibility of these measurements. Secondly, previous studies have shown that subsequent light sensitivity varies with prior light history [40,41,42]. In the present study, light exposure prior to laboratory entry was monitored with an activity meter but was not controlled. No significant correlation was found between the log average illuminance of light exposure on the day of laboratory entry and the amount of light-induced DLMO advance (r = 0.20), but future studies are needed to determine the effect of prior light history on the light-induced phase shift of circadian rhythms. Furthermore, in this study, light exposure was conducted after confirming phase delay under dim light, and the subjects therefore spent about 40 **h** in an environment with insufficient light prior to light exposure. This is an environment that is not common in real life, and it is possible that the dim environment increased subjects’ photosensitivity and that the effects of light exposure were overestimated.

In conclusion, our findings suggest that the ease of phase delay of an individual’s circadian phase is related to the magnitude of the phase advance due to morning light. As a study that followed a short-term phase shift, the results of this study can be applied to real life. In recent years, various health risks caused by nighttime light exposure have been reported [43,44,45]. The results of this study suggest that even without nighttime light exposure, light deprivation during the day may cause phase delay even for a single day as previous studies showed [5,6,7,10]. However, the phase was reset by one hour of light exposure the following morning, emphasizing the importance of a certain amount of intense light expo-sure in the morning such as sunlight (e.g., commuting to work or school, taking a walk). The amount of natural phase delay in this study was about 15 min on average, and there is no evidence that this is a direct health risk. However, the finding that this is related to the amount of light-induced phase advance is expected to contribute to the establishment of a better light environment that is tailored to individual differences in circadian phase shifts.

## 4. Materials and Methods

### 4.1. Subjects

Table 2 shows the characteristics of the subjects who participated in this study and whose data were used in the analysis. Twenty-eight healthy male and female undergraduate and postgraduate students (twelve men and sixteen women, mean age: 22.2 ± 2.3 years) participated in the study and one individual was excluded from the analysis. According to the Morningness-Eveningness Questionnaire (MEQ) [46,47], there were no extreme morning or evening types. Subjects were asked not to travel across time zones for two weeks prior to participation in the study. Signed written informed consent, which was approved by the Ethical Committee of Kyushu University, was obtained from all participants. The experiments were conducted in accordance with the Declaration of Helsinki.

### 4.2. Experimental Conditions

The vertical illuminance of each light condition was measured at eye level in a sitting position using an illuminance spectrophotometer (CL-500A, Konica Minolta, Tokyo, Japan), and melanopic EDI [48] was calculated as the quantity that accounts for the non-visual effects of light. Subjects spent the daytime in a dim room (~3 lx) with voltage-adjustable incandescent bulbs in four corners of the room. Although staring at the light source was prohibited, they encountered a range of illuminance (2.3–3.5 lx) as they could look in various directions. Light exposure took place in a bright room (8000 lx 4100K, 4951 lx (melanopic EDI)) that was equipped with RGB fluorescent lamps on the ceiling. The alpha-opic illuminance of the dim light and that of the bright light and the spectral distributions of both lights are shown in Table S1 and Figure S2, respectively. During the bright light exposure, each subject’s head was fixed on a chinrest and a small monitor was placed horizontally 70 cm away from the head. The subjects were instructed to gaze at the video on the small monitor and not to move. Subjects slept in a dark room (<0 lx) with no light. Each room was set to a temperature of 25.5 °C and humidity of 50%.

### 4.3. Procedure

The experiment was conducted during the period from August to September in 2022. The subjects were instructed to live freely as usual for two weeks before entering the laboratory but were prohibited from staying up excessively late or staying up all night, which they did not do in their daily routine. They reported sleep onset time and wake time immediately after getting up every day online. Sleep was checked using an activity meter with an illuminance meter (Motion Watch 8, CamNtech, Cambridge, UK). The subjects were asked not to consume excessive caffeine or alcohol and not to smoke many cigarettes for two weeks before the start of the laboratory experiment. They were also instructed not to take sleeping pills or other drugs that cause sleepiness. The subjects participated in a three-night (Day 1 to Day 3) laboratory experiment to examine phase shifts in circadian rhythm. Caffeine and alcohol consumption were prohibited even in small quantities on the day the subjects entered the laboratory.

**Figure 4:**
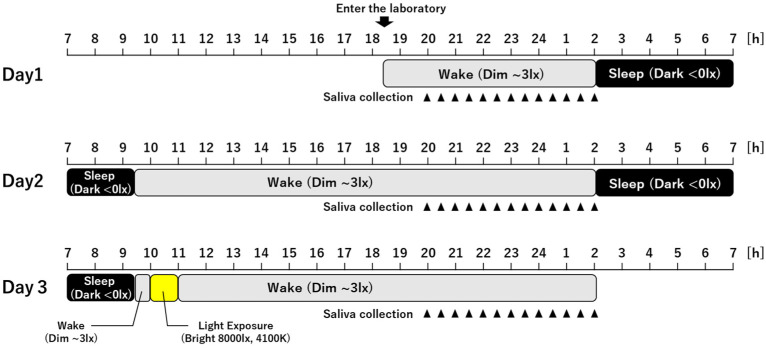
Example of the experimental protocol setup to take into account the predicted DLMO with reference to MSFsc. This figure shows an example in the case of predicted DLMO = 23:00. This schedule was shifted in 30 min increments according to the individual’s predicted DLMO.

The flow of the experiment is shown in Figure 4. Subjects arrived at the laboratory on the evening of the first day of the experiment and they received a briefing and entered the dim room. The experimental schedule was shifted in 30 min increments according to the subject’s estimated DLMO. The individual DLMO phase was estimated by calculating the midpoint of sleep on free days corrected for sleep debt accumulated through weekdays (MSFsc) calculated from the subjects’ sleep habits in the Munich chronotype questionnaire (MCTQ) [49,50]. DLMO was estimated by the formula created from the relationship between DLMO and MSFsc in previous studies (DLMO = 0.468 × MSFsc + 20.2, unpublished data). Based on the estimated DLMO, six hours of saliva collection time was set. Subjects collected saliva every 30 min in the dim room at the same time during the night on all days. Saliva samples were collected using swabbed tubes (Salivette, Sarstedt, Germany). Participants did not drink water for 10 min before each saliva sample was taken. After saliva collection, participants started to sleep 3 h after predicted DLMO and slept for 7.5 h in the dark room. The next morning, they entered the dim room after waking up again. On Day 3, participants moved to the dim room after waking and were exposed to bright light in the bright room for 1 h starting 30 min after waking and then returned to the dim room. Light exposure was set to begin 11 h after the predicted DLMO, when a stable phase advance independent of the circadian phase due to light exposure could be expected, based on the PRC of a previous study using light conditions similar to those in this study [19]. In this study, subjects went to bed 3 h after the predicted DLMO and woke up 10.5 h later. As a result, the time in bed was fixed at 7.5 h. The reason for this is that the protocol for this experiment was designed to ensure successful measurement of DLMO and the timing of morning light exposure to be carried out 11 h after predicted DLMO. Thus, it was not possible to time-schedule the laboratory experiments based on the subjects’ usual sleep habits and the timing did not necessarily coincide with individuals’ habitual bedtime and wake times. The subjects’ bedtime and wake time in the laboratory were 2:01 ± 0:41 and 9:31 ± 0:41, respectively, in clock time. These showed a high consistency with sleep onset time (sleep onset weekday: r = 0.84, sleep onset free day: r = 0.96) and wake time (wake time weekday: r = 0.77, wake time free day: r = 0.85) in MCTQ coefficients. Given that, on average, this experimental schedule was similar to the subjects’ schedules on free days and was in accordance with some extent with real life, it is likely that the results of this study would not have changed even if the laboratory experiments could have been scheduled according to the subjects’ sleep habits.

Up to three subjects were tested simultaneously in the same room. The subjects were allowed to read, play board games, and use laptops, smart phones and game consoles in the dim room. When using electronic devices, the screen illumination was set to the lowest luminance so as not to affect the circadian phase shift, and if the illuminance in the screen direction exceeded 5 lx, a smoke sheet was placed over the screen to ensure that the illuminance in front of the eyes was at the set illuminance (~3 lx). The subjects were also prohibited from eating freely, napping, and exercising except for short periods of stretching during the experiment. Meals for the subjects were similar (typical Japanese food) and were given three times a day on Day 2 and Day 3. Except for the saliva collection time, the subjects were free to drink water, but no drinks other than water were offered during the experiment. The subjects were prohibited from drinking water from 10 min prior to saliva collection until the time of saliva collection and they remained at rest. They were allowed to use the restroom freely except for the time of rest in the dim light and the time of light exposure. They were also allowed to shower prior to the start of saliva collection on each day. Both the restroom and shower room had the same light intensity (dim) settings as those in the laboratory.

### 4.4. Sample Analysis

The saliva samples were centrifuged, immediately frozen at −30 °C, and stored until analysis. The salivary melatonin concentration was then measured by using a radioimmunoassay kit (RK-DSM2-U, NovoLytiX, Switzerland). Linear interpolations were made from the salivary melatonin concentration at each time on each day. Dim light melatonin onset (DLMO) [51], a marker of circadian phase, was determined as the time when the salivary melatonin concentration first exceeded 3.0 pg/mL and DLMO for each day was identified. The amount of DLMO shift was calculated from the difference between the DLMOs on each day. DLMO shift from Day 1 to Day 2 was calculated as natural phase delay under dim light (phase shift 1; PS1) and DLMO shift from Day 2 to Day 3 was calculated as phase advance due to the morning light exposure (phase shift 2; PS2). Phase angles were calculated as the time difference from the DLMO of Day 1 to each of the sleep variables in the MCTQ. The circadian time of light exposure (start time) was calculated as the relative time from the DLMO of Day 2 to the clock time when the light started.

### 4.5. Statistical Analysis

Twenty-seven individuals were used in the analysis. One individual who was an outlier in PS1 was excluded from the analysis. The relationship between PS1 and PS2 was also examined. The relationships of chronotype-related indicators (Day 1 DLMO as circadian phase, chronotype as MEQ score, sleep habits in MCTQ, phase angles) with PS1 and PS2 were also examined. For multiple correlation analysis, false discovery rate (FDR) correction was used. A paired *t*-test was used to test the significance of the DLMO shifts and Welch’s *t*-test was used to test the sex difference of Day1 DLMO and DLMO shifts. Pearson’s correlation analysis or partial correlation analysis was used for the relationships between indices. When examining correlation coefficients or conducting *t*-tests, the Kolmogorov–Smirnov test was used beforehand to confirm the normality of the data before analysis. A *p*-value less than 0.05 was considered statistically significant and FDR was set to 0.05. These analyses were carried out with SPSS ver. 25 or R 4.2.2.

## Figures and Tables

**Table 1:** Correlations between phase shifts and chronotype-related indicators.

		Mean	SD	Pearson’s r with PS1	Pearson’s r with PS2
	Day 1 DLMO	22:17	1:26	−0.21	0.47
	MEQ score	47.6	9.6	−0.23	−0.18
Weekday	Sleep Onset	1:11	1:06	−0.14	0.28
	Phase Angle [h]	2.91	1.02	0.15	−0.36
	Midsleep	4:43	1:04	0.01	0.16
	Phase Angle [h]	6.45	1.06	0.30	−0.48
	Wake	8:15	1:09	0.15	0.03
	Phase Angle [h]	9.98	1.21	0.40	−0.53
	Sleep Duration [h]	7.06	0.75	0.44	−0.36
Free day	Sleep Onset	1:55	1:25	−0.09	0.16
	Phase Angle [h]	3.64	1.19	0.15	−0.39
	Midsleep	5:42	1:21	0.04	0.13
	Phase Angle [h]	7.43	1.16	0.31	−0.44
	Wake	9:30	1:27	0.16	0.08
	Phase Angle [h]	11.22	1.34	0.40	−0.42
	Sleep Duration [h]	7.59	0.98	0.37	−0.11
	MSFsc	5:33	1:22	0.04	0.09
	Social Jetlag [h]	0.98	0.69	0.06	0.00

n = 27, Phase angles indicate time difference from DLMO of Day 1 to sleep variables.

**Table 2:** Characteristics of subjects in this study.

	Mean	SD
Age [years]	22.2	2.3
Weight [kg]	53.7	8.7
Height [cm]	164.8	9.5
BMI [kg/m^2^]	19.7	2.0

n = 27, twelve males and fifteen females.

## Data Availability

The datasets generated from the study are available from the corresponding author on reasonable request.

## Supplementary Materials

The following supporting information can be downloaded at: https://www.mdpi.com/article/10.3390/clockssleep5040041/s1, Figure S1: Individual melatonin concentration profiles used in the analysis (n = 27), Figure S2: Spectral distributions of dim light (a) and bright light (b), Table S1: Illuminance, correlated color temperature and alpha-opic illuminance of light conditions in this study.
