# Supplementary material for: Relationship between Circadian Phase Delay without Morning Light and Phase Advance by Bright Light Exposure the Following Morning
Source: Clocks Sleep. 2023 Oct 23;5(4):615–26. doi: 10.3390/clockssleep5040041 (PMC10594521; doi:10.3390/clockssleep5040041)
Supplement: Supplementary file 1 [file clockssleep-05-00041-s001.zip › clockssleep-2561347-supplementary/TableS1.pdf]

Table S1. Illuminance, correlated color temperature and alpha-opic illuminance of light conditions in this study

	Photopic illuminance [lx]	Correlated color temperature [K]	Alpha-opic equivalent daylight (D65) illuminance [lx]				
			S-cone-opic	M-cone-opic	L-cone-opic	Rhodopic	Melanopic
Dim light	2.6	2752	0.6	2.1	2.7	1.4	1.2
Bright light	8004	4103	4989	7054	7966	5506	4951

All measurements were performed vertically at the sitting subject's eye level.
