# Supplementary figures and images for: Relationship between Circadian Phase Delay without Morning Light and Phase Advance by Bright Light Exposure the Following Morning
Source: Clocks Sleep. 2023 Oct 23;5(4):615–26. doi: 10.3390/clockssleep5040041 (PMC10594521; doi:10.3390/clockssleep5040041)

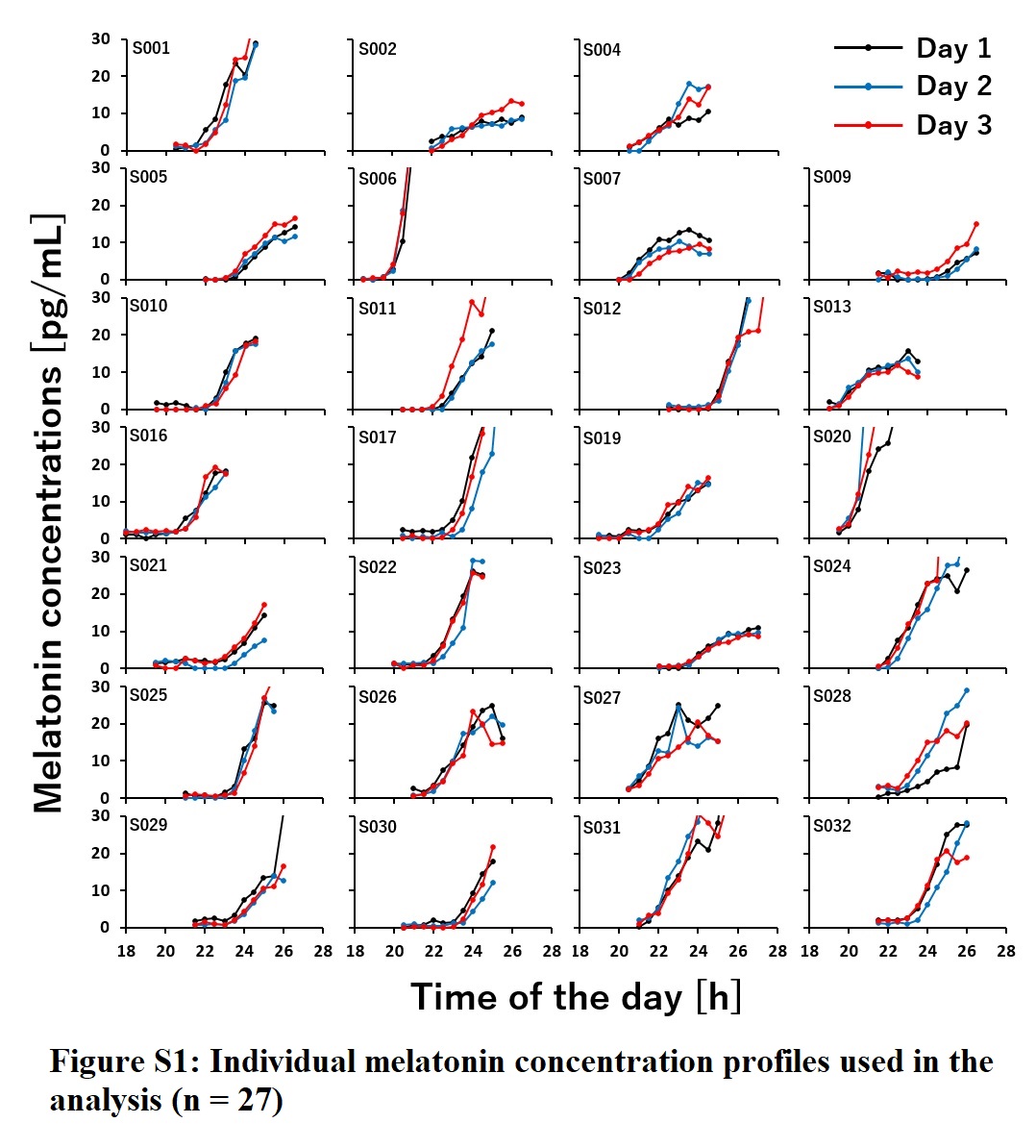

Supplement: Supplementary file 1 [file clockssleep-05-00041-s001.zip › clockssleep-2561347-supplementary/FigureS1.jpg]

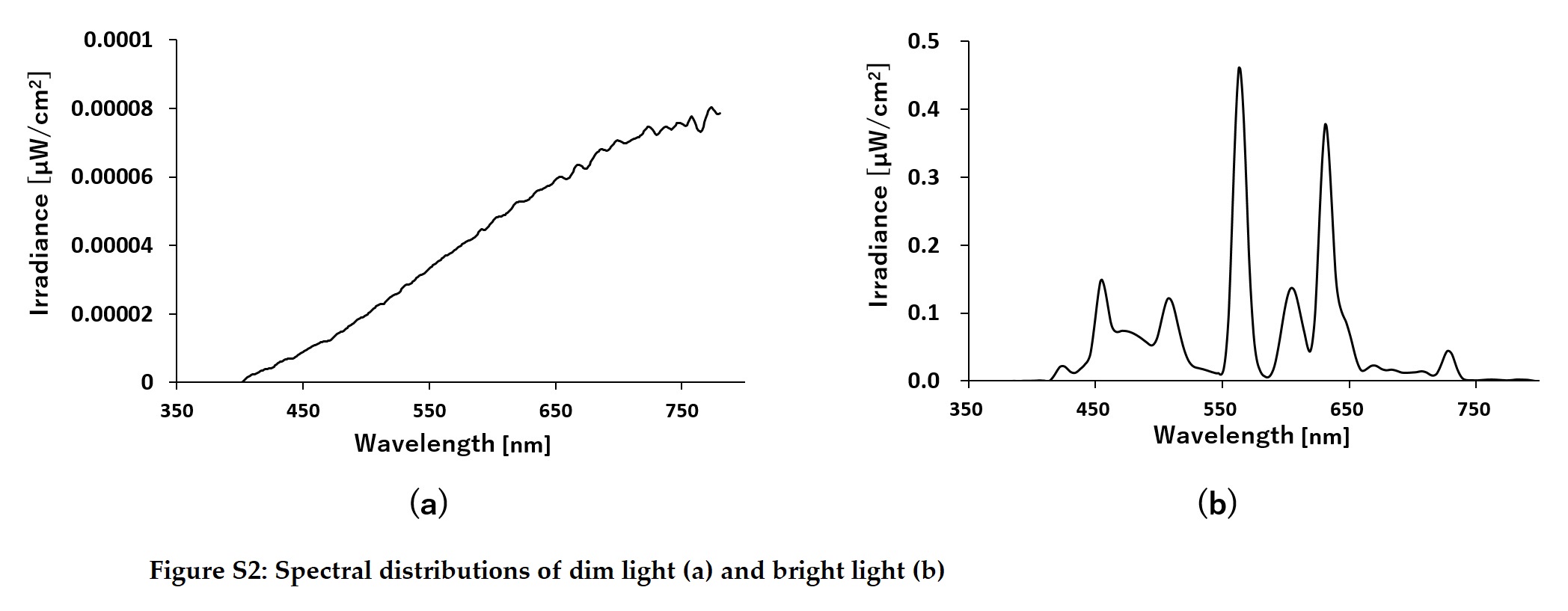

Supplement: Supplementary file 1 [file clockssleep-05-00041-s001.zip › clockssleep-2561347-supplementary/FigureS2.jpg]
